# Pesticide Testing on Humans: Resnick and Portier Respond

**Published:** 2005-12

**Authors:** David B. Resnik, Christopher Portier

**Affiliations:** Office of the Scientific Director, National Institute of Environmental Health Sciences, National Institutes of Health, Department of Health and Human Services, E-mail: resnikd@niehs.nih.gov; National Toxicology Program, National Institute of Environmental Health Sciences, National Institutes of Health, Department of Health and Human Services

In their letter, Needleman et al. suggest that our arguments regarding the ethics of human testing of pesticides (Resnik and Portier 2005) are “vague, tendentious, and essentially incorrect.” However, they offer no effective sustained arguments in support of this conclusion.

They cite the Food Quality Protection Act (FQPA 1996) and note that this act led pesticide companies to sponsor studies in humans, in part, to avoid an additional 10-fold human safety factor. They then chastise our article for saying effectively the same thing. Their next point focuses on what they view as a U.S. Environmental Protection Agency (EPA) failure to enforce the FQPA for organophosphate pesticides, an issue that has no bearing on the ethical question of human testing of pesticides. They claim that we failed to specify exactly how human studies could benefit society, but we clearly stated in our article (Resnik and Portier 2005) that these studies could promote public health by providing knowledge that may be useful in regulating pesticides. It is the responsibility of the party sponsoring or conducting a human testing study to demonstrate the relevance and utility of the proposed study with regard to human toxicity.

Needleman et al. contend that we have missed an important issue—the relevance of adult testing to children’s risk. This argument is not relevant to our article because we focused on human testing of pesticides on adults, not on children. We seriously doubt whether testing pesticides on children, or on other vulnerable populations, could ever be justified on ethical grounds. It is possible that studies of the effects of pesticides on adults could enhance our understanding of how pesticides affect children, but the party(s) sponsoring the study would need to provide some evidence for this supposition. In our article (Resnik and Portier 2005) we argued that there may be some cases where the public health benefits of testing pesticides on adults justify imposing risks on human subjects. We did not argue that one of the benefits of testing pesticides on adults is to improve the health of children, but we acknowledge that some studies might have this potential benefit.

We addressed arguments concerning the statistical power of human studies in our Supplemental Material, which is available online (http://ehp.niehs.nih.gov/members/2005/7720/suppl.pdf). Good statistical design is one of the key principles that must be considered in evaluating the acceptability of human testing, and we acknowledge that some of the disputed pesticide studies have been underpowered. It is surprising to us that Needleman et al. would raise this concern when their key premise is that human testing of pesticides is never ethical. If testing of pesticides is never ethical, then statistical issues, such as sample size, are irrelevant.

Needleman et al. have confused the issues relating to the FQPA, 10X safety factors, and risk assessment with the questions surrounding human testing of pesticides. We wholeheartedly agree that to conduct a clinical study in humans for the sole purpose of keeping a product commercially viable is unethical. They assume that the only reason why anyone would develop and conduct human studies of pesticides is to promote the interests of pesticide manufacturers. Again, we address this issue in our Supplemental Material (http://ehp.niehs.nih.gov/members/2005/7720/suppl.pdf).

We argue that a study that benefits private industry can be ethical, provided that it also offers scientific or social benefits. For example, clinical trials of new drugs benefit pharmaceutical companies, but they also benefit patients, enhance our understanding of human disease, and improve public health.
